# Supplementary material for: The causal role of circulating inflammatory markers in osteoporosis: a bidirectional Mendelian randomized study
Source: Front Immunol. 2024 Jul 18;15:1412298. doi: 10.3389/fimmu.2024.1412298 (PMC11291241; doi:10.3389/fimmu.2024.1412298)
Supplement: Supplementary file 1 [file DataSheet_1.zip › supplementary material/OSTEOPOROSIS-CIM/GCST90274785.tsv.gz_forest.pdf]

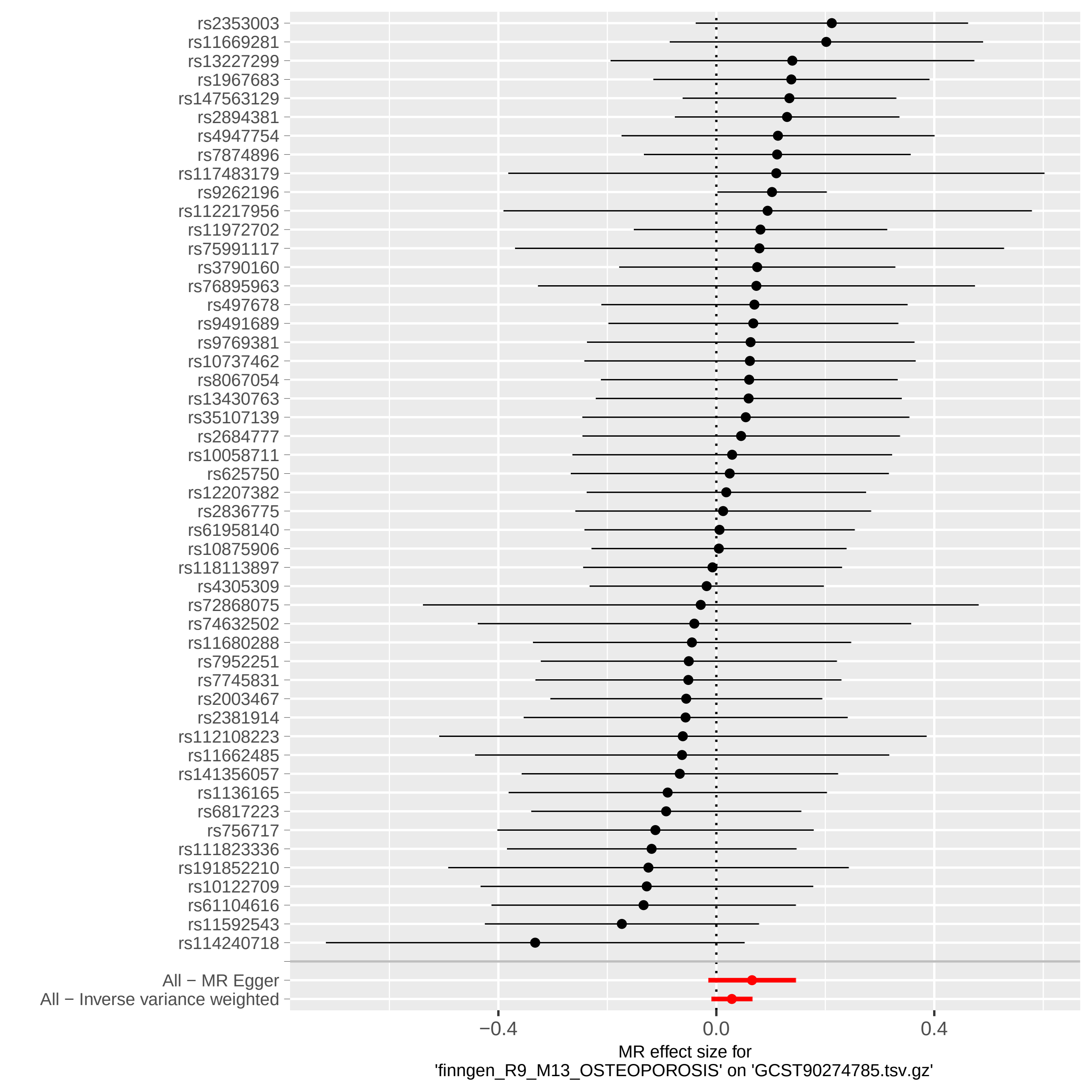
rs2353003
rs11669281
rs13227299
rs1967683
rs147563129
rs2894381
rs4947754
rs7874896
rs117483179
rs9262196
rs112217956
rs11972702
rs75991117
rs3790160
rs76895963
rs497678
rs9491689
rs9769381
rs10737462
rs8067054
rs13430763
rs35107139
rs2684777
rs10058711
rs625750
rs12207382
rs2836775
rs61958140
rs10875906
rs118113897
rs4305309
rs72868075
rs74632502
rs11680288
rs7952251
rs7745831
rs2003467
rs2381914
rs112108223
rs11662485
rs141356057
rs1136165
rs6817223
rs756717
rs111823336
rs191852210
rs10122709
rs61104616
rs11592543
rs114240718
All − MR Egger
All − Inverse variance weighted
−0.4
0.0
0.4
MR effect size for
'finngen_R9_M13_OSTEOPOROSIS' on 'GCST90274785.tsv.gz'
